# Urinary-based detection of MSL, HE4 and CA125 as an additional dimension for predictive and prognostic modelling in ovarian cancer

**DOI:** 10.3389/fonc.2024.1392545

**Published:** 2024-07-15

**Authors:** Franziska Maria Schwarz, Daniel Martin Klotz, Pauline Wimberger, Jan Dominik Kuhlmann

**Affiliations:** ^1^ Department of Gynecology and Obstetrics, Medical Faculty and University Hospital Carl Gustav Carus, Technische Universität Dresden, Dresden, Germany; ^2^ National Center for Tumour Diseases (NCT), Dresden, Germany: German Cancer Research Center (DKFZ), Heidelberg, Germany; Faculty of Medicine and University Hospital Carl Gustav Carus, Technische Universität Dresden, Dresden, Germany; Helmholtz-Zentrum Dresden-Rossendorf (HZDR), Dresden, Germany; ^3^ German Cancer Consortium (DKTK), Dresden and German Cancer Research Center (DKFZ), Heidelberg, Germany

**Keywords:** ovarian cancer, urine, MSL, CA125, HE4, prognostic modelling

## Abstract

**Objectives:**

We have recently described a predictive/prognostic model for ovarian cancer, exploiting commonly available clinico-pathological parameters and the ovarian serum biomarkers mesothelin (MSL), human epididymis protein 4 (HE4) and cancer-antigen 125 (CA125). Considering urine as a prototype non-invasive sample, we investigated whether serum levels of these biomarkers are mirrored in urine and compared their clinical relevance in matched serum *vs.* urine samples.

**Methods:**

MSL, HE4 and CA125 were quantified in urinary (n=172) and matched serum samples (n=188) from ovarian cancer patients (n=192) using the Lumipulse^®^ G chemiluminescent enzyme immunoassay (Fujirebio).

**Results:**

While absolute concentrations of MSL or CA125 were higher in serum than in matched urine samples, HE4 concentrations were considerably higher in urine than in serum. Nonetheless, the levels of all three biomarkers strongly correlated between matched serum *vs.* urine samples and were unrelated to *BRCA1/2* mutational status. Consequently, prediction of surgical outcome or relapse/death by MSL, HE4 or CA125 was similarly efficient among urinary- *vs.* serum-based detection. HE4 provided the highest capacity to predict surgical outcome or relapse/death among both body fluids (urine: AUC=0.854*;* serum: AUC=0.750, respectively). All clinically relevant findings regarding the investigated urinary biomarkers were equally reproducible among raw *vs.* creatinine-normalized datasets, suggesting that normalization may have subordinate priority for urine-based analysis of these biomarkers.

**Conclusion:**

We report that the capacity of MSL, HE4 and CA125 to predict surgical outcome and relapse/death is equivalent between serum *vs.* urine-based detection. Urinary biomarkers, in particular HE4, may provide an additional dimension for prognostic modeling in ovarian cancer.

## 1 Introduction

Ovarian cancer is the leading cause of death for women with gynecological malignancies and more than 70% of patients are diagnosed at already advanced FIGO stages (1). Standard treatment of advanced ovarian cancer consists of surgical debulking, aiming at macroscopic complete tumor resection, and platinum/paclitaxel-based chemotherapy. Despite advanced primary therapy, such as maintenance treatment with the anti-angiogenic antibody bevacizumab or the use of PARPi inhibitors in patients with homologous repair deficient (HRD) tumors (2–4), still the majority of advanced ovarian cancer patients experience relapse (5, 6). Considering the poor overall prognosis of ovarian cancer, the identification of non-invasive predictive/prognostic biomarkers is of high clinical interest.

In the last years, numerous prognostic models for ovarian cancer have been proposed, extending from single serum parameters to gene-expression profiling (7, 8). However, none of these models have been translated into clinical practice. We have recently described a robust predictive and prognostic model for ovarian cancer by exploiting commonly available clinico-pathological factors and three ovarian cancer serum biomarkers. These included i) mesothelin (MSL), a glycosyl-phosphatidyl inositol-anchored membrane glycoprotein ii) human epididymis protein 4 (HE4), a glycoprotein commonly expressed in ovarian tumors and iii) cancer antigen 125 (CA125), the current gold standard tumor marker in ovarian cancer. We reported that detection of pre-operative serum HE4 and CA125 was the optimal marker combination for blood-based prediction of surgical outcome (AUC=0.86) (9). Moreover, we selected a minimal set of clinico-pathological risk factors and/or serum parameters for constructing a robust prognostic model for ovarian cancer patients. In this regard, detection of three serum parameters (pre-operative CA125, post-operative MSL and HE4), in addition to the surgical outcome status, resulted in optimal prediction of survival. Prognostic performance of our model was superior to any of the investigated parameters alone and was independent from *BRCA1/2* mutational status. We subsequently transformed this model into a prognostic risk index, predicting relapse or death with an AUC of 0.89 (9).

Urine as the prototype non-invasive sample is a highly attractive fluid for biomarker discovery, since it is easily accessible without any trauma or pain on the patients and can be collected longitudinally and in a quantitative manner. Compared with serum or plasma, urinary peptides have several advantages for biomarker discovery, such as a high content of small and stable molecules. Furthermore, the urinary proteome is supposed reflect an informative “end product” of the abnormal protein metabolism in cancer (10), which is an ideal condition for the discovery of cancer specific biomarkers. In the last years, a variety of potential urinary biomarkers have been proposed for ovarian cancer, including individual proteins (MSL, HE4, CA125, BCL-2), microRNAs (miR-30a-5p, miR-6076) or metabolites (polyamines, succinate) (11). As these previous studies focused on early diagnosis, the clinical utility of urinary biomarkers for prognostic modelling in ovarian cancer is still an open question and comparative studies on matched serum *vs.* urine samples for outcome prediction are mostly lacking.

The objective of this study was to investigate, whether our previously described predictive/prognostic model (9) can successfully be transferred from a serum- to a urinary-based detection platform. In detail, we investigated whether serum levels of MSL, HE4 and CA125 are mirrored in urine and comparatively analyzed their clinical relevance to predict surgical outcome and prognosis in matched serum *vs.* urine samples. Finally, we defined the minimally required biomarker setting for optimal urinary-based outcome prediction.

## 2 Patients and methods

### 2.1 Patient characteristics

Patients were recruited and samples were obtained at the Department of Gynecology and Obstetrics at the Technische Universität Dresden, Germany with inclusion criteria as described before (9). Overall, 192 consecutive patients with histologically confirmed primary epithelial ovarian cancer and a primary diagnosis from 2013-2020 were included into our retrospective study. According to national guidelines, patients received cytoreductive surgery with the aim of macroscopic complete tumor resection, which was followed by platinum-based adjuvant chemotherapy. Patients treated with primary or neoadjuvant chemotherapy followed by interval debulking surgery were excluded. Participation in clinical trials did not result in exclusion of a patient. Overall survival (OS) and progression-free survival (PFS) was calculated from the date of primary diagnosis. The study had been approved by the Local Research Ethics Committee at the Technische Universität Dresden, Germany (file number: EK74032013) and was performed in accordance with good clinical practice guidelines, the Declaration of Helsinki and national laws. All study participants gave written informed consent. Patients’ clinical data are summarized in **Table 1**. Ovarian cancer was reported in agreement with the WHO‐classification of tumors derived from female genital tract and staging was documented according to the FIGO classification (12), revised in 2014 (13). FIGO-stage was reported according to the revised version for all patients, who underwent primary surgery from 2014 onwards. In the case of no contraindications, patients with a tumor stage of FIGO III-IV were additionally treated with the antiangiogenic antibody bevacizumab in parallel to chemotherapy, which was followed by bevacizumab maintenance therapy. Surgical outcome was reported dichotomously (macroscopically complete tumor resection *vs.* any residual tumor).

**Table 1:** Patient characteristics at primary diagnosis.

**Age**	**Median (range)**	64 years (23 - 82 years)	
**FIGO stage**	**I/II**	33	19.2%
	**III/IV**	139	80.8%
**Surgical debulking**	**Any residual tumor**	71	41.3%
	**Macroscopic complete resection**	101	58.7%
**Histology**	**Serous**	154	89.5%
	**Non-serous**	18	10.5%
**BMI**	**BMI >= 30**	128	74.4%
	**BMI <30**	44	25.6%
***BRCA1/2*** **mutational status**	***BRCA1/2wt***	75	43.6%
	***BRCA1/2mut***	31	18.0%
	**Unknown**	66	38.4%
**Progression-free survival**	**Median** (range)	20.3 months (0.8 – 87.6 months)	
	**Progression/death**	86	50%
	**No progression/death**	86	50%
**Overall survival**	**Median** (range)	31 months (0.8 - 88.8 months)	
	**Dead**	56	32.6%
	**Alive**	116	67.4%
**MSL levels**	**Median** (interquartile range)		
	**Urine**	0.24 nmol/L (0.05 – 1.06 nmol/L)	
	**Serum**	1.57 nmol/L (0.88 – 3.87 nmol/L)	
**HE4 levels**	**Median** (interquartile range)		
	**Urine**	35507.3 pmol/L (10235.3 - 127729.3 pmol/L)	
	**Serum**	479.3 pmol/L (162.9 – 973.6 pmol/L)	
**CA125 levels**	**Median** (interquartile range)		
	**Urine**	11.55 U/mL (3.9 – 47.5 U/mL)	
	**Serum**	401.1 U/mL (76.9 – 944.3 U/mL)	

### 2.2 Urine preparation

Urine preparation was performed from pre-operative urine samples obtained in a 7.5 ml S‐Monovette^®^ (Sarstedt AG & Co., Nuembrecht, Germany). The urine was prepared by centrifugation for 8 min at 1,800 g at room temperature and was immediately frozen at -80°C until further processing with avoidance of unnecessary freeze-thaw cycles. For analysis, samples were thawed on ice and were immediately processed after complete thawing. Samples were blinded to the laboratory staff during processing.

### 2.3 Serum preparation

Serum preparation was performed from pre-operative blood-samples as described previously (9). In brief, blood was withdrawn with a 7.5 ml S‐Monovette^®^ (Sarstedt AG & Co., Nuembrecht, Germany), followed by an incubation at room temperature for at least 30 min to allow complete blood coagulation. Within 1 h after blood drawing, serum was prepared by centrifugation for 8 min and 1,800 g at room temperature. The isolated cell free serum fraction was immediately frozen at -80°C until further processing with avoidance of unnecessary freeze-thaw cycles. For analysis, samples were thawed on ice and were immediately processed after complete thawing. Samples were blinded to the laboratory staff during processing.

### 2.4 Detection of CA125, HE4 and MSL

Urine levels of CA125, HE4 and MSL were quantified using fully automated chemiluminescent immunoassays (Lumipulse^®^ G CA125-II, G HE4, G SMRP). All steps were performed on a LUMIPULSE G1200 analyzer according to the manufacturer’s instructions (Fujirebio Europe, Gent, Belgium). Corresponding serum values of these markers were determined by the same method as described previously (9). The analytic sensitivity for CA125, HE4 or MSL detection were 2.0-1000 U/mL, 20-1500 pmol/L and 0.1-100 nmol/L respectively. The urine specimens for HE4 measurement were pre-diluted 1/100 using the Lumipulse Specimen Diluent.

### 2.5 Statistical analysis

Statistical analysis was conducted with GraphPad Prism version 10.1.2 (GraphPad Software, La Jolla, CA, USA), adapted from (14, 15) and with Addinsoft (2022) XLSTAT statistical and data analysis solution (New York, USA). P‐values <0.05 were considered statistically significant. All confidence intervals (CIs) were specified as 95%CI. Differences of the median were calculated by the Mann-Whitney test or Kruskal-Wallis test with Dunn’s correction for multiple comparisons. Principal component analysis (PCA) was performed by Addinsoft (2022) XLSTAT statistical and data analysis solution (New York, USA). Pearson correlation and linear regression were used to assess correlations of the matched samples. Urinary biomarker cut-off levels were established using receiver operating characteristic (ROC)-curve analysis. Kaplan-Meier analyses were performed with significance levels indicated by log-rank (Mantel-Cox) analysis. Multivariate logistic regression analysis for the prediction of PFS (relapse/death) before 48 months were adjusted for established risk factors of ovarian cancer, such as age, BMI or surgical outcome. The best marker combination was selected using likelihood and Akaike-Information-Criterion (AIC) assessment (selection of lowest AIC-value) from the considered biomarkers.

## 3 Results

### 3.1 Correlation of MSL, HE4 and CA125 levels in matched serum *vs.* urine samples

**Figure 1:**
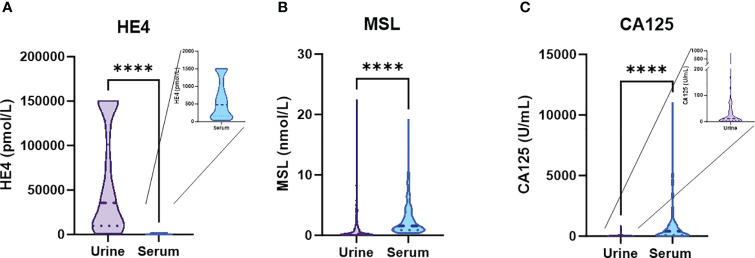
Absolute concentrations of the designated biomarkers in urine *vs.* serum. Violin plots depicting absolute concentrations of **(A)** HE4, **(B)** MSL and **(C)** CA125 in matched urine *vs.* serum samples. The dashed line depicts the median and the dotted lines the first and third quartiles. P-values according to the Mann-Whitney test are indicated. ****P<0.0001.

We quantified MSL, HE4 and CA125 in pre-operative urine samples in a retrospective cohort of n=192 clinically documented ovarian cancer patients using Lumipulse^®^ G chemiluminescent enzyme immunoassays and correlated these data with matched serum values of each parameter, as determined previously (9). Comparing the absolute concentrations between urine *vs.* serum, we observed that the biomarker with lowest molecular weight of 25 kDa (HE4) was considerably higher concentrated in urine than in serum (estimated difference (ED)= 59733 pmol/L, p<0.0001; **Figure 1A**). This suggests that HE4 efficiently passes the glomerular filter of the kidneys. MSL as a larger molecule (40 kDa) was lower concentrated in urine than in serum (ED=3 nmol/L, p<0.0001; **Figure 1B**). CA125, with a considerably higher molecular weight (110 kDa), was present near detection limit in urine samples and was around 1000-fold higher concentrated in matched serum samples (ED= 1085 U/mL, p<0.0001; **Figure 1C**). This was consistent with the fact that serum HE4 but not serum CA125 correlated with serum creatinine (sCREA), a marker of kidney function (r=0.2484, p=0.0008; r=0.0673, p=0.3710, respectively).

**Figure 2:**
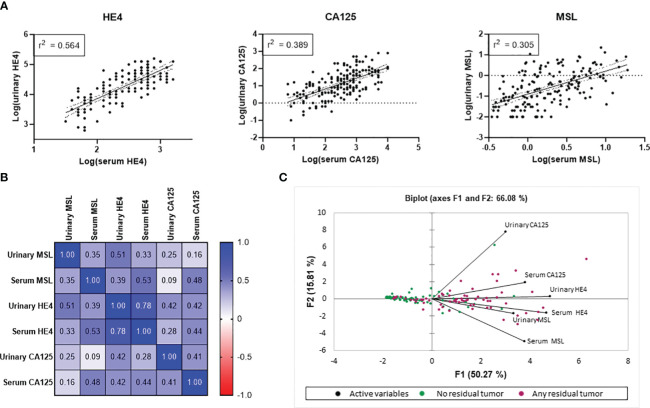
Correlation of HE4, CA125 and MSL in urine *vs.* serum. Correlation metrics of the three biomarkers are reported according to **(A)** linear regression analysis and **(B)** Pearson correlation. **(C)** Principle Component analysis (PCA) combining all investigated parameters. Patients with any residual tumor are indicated by red data points. Patients with macroscopic complete resection are indicated by green data points.

From our patient cohort, matched values of urine *vs.* serum measurements were available in n=172/192 patients for MSL, in n=103/192 patients for HE4 and n=171/192 patients for CA125. According to linear regression analysis, there was a correlation between urine *vs.* serum levels for all three parameters, which was strongest for HE4 (r^2^ = 0.564), followed by CA125 (r^2^ = 0.389) and MSL (r^2^ = 0.305; **Figure 2A**). This finding was corroborated by a Pearson correlation matrix, reporting significant correlations for the three parameters, again with HE4 showing strongest correlation between urine *vs.* serum (HE4: r=0.784; CA125 r=0.406, MSL r=0.352; **Figure 2B**). Consistently, principle component analysis (PCA) revealed that the closest associations among the comparison between serum *vs.* urinary biomarkers was evident for HE4. Considering all biomarkers, the closest association was observed between serum HE4 and urinary MSL (**Figure 2C**).

We conclude that, based on their molecular weight, the investigated biomarkers showed crucial differences in their absolute urinary *vs.* serum concentrations, indicating different glomerular filtration rates. Nonetheless, levels of all three biomarkers strongly correlated between matched serum vs. urine samples, with HE4 showing the most stringent correlation.

### 3.2 Association of urinary MSL, HE4 and CA125 levels with the patients’ clinicopathological parameters

**Figure 3:**
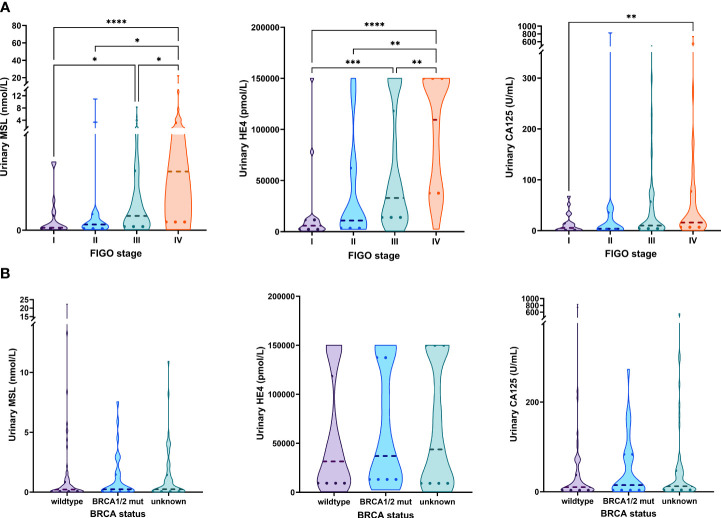
Association of MSL, HE4 and CA125 levels with FIGO-stage and *BRCA* status. Comparison of MSL, HE4 or CA125 levels values across **(A)** the different FIGO-stages **(B)** *BRCA1/2* status. The dashed line depicts the median and the dotted lines the first and third quartiles. Adjusted P-values according to the Kruskal-Wallis test with Dunn’s correction are indicated. *P<0.05, **P<0.01, ***P<0.001, ****P<0.0001.

We observed a strong association between all three investigated biomarkers and FIGO-stage, with significantly elevated levels of each biomarker between FIGO I *vs.* FIGO IV. Notably, HE4 exhibited the most pronounced gradual increase among the different FIGO-stages (**Figure 3A**). From all patients with an available pre-operative urine sample, 106/172 had a known *BRCA1/2* mutational status. Of those, 75 patients (71%) were *BRCA1/2* wild type (*BRCA1/2*wt), whereas 31 patients (29%) had a pathogenic somatic or germline *BRCA1/2* mutation (*BRCA1/2*mut). Median levels of urine MSL, HE4 or CA125 did not significantly differ between *BRCA1/2*wt *vs. BRCA1/2*mut patients (**Figure 3B**).

Taken together, urinary levels of MSL, HE4 and CA125 paralleled FIGO-stage but were unrelated to the patient’s *BRCA1/2* mutational status.

### 3.3 Urinary- *vs*. serum-based prediction of surgical outcome

**Figure 4:**
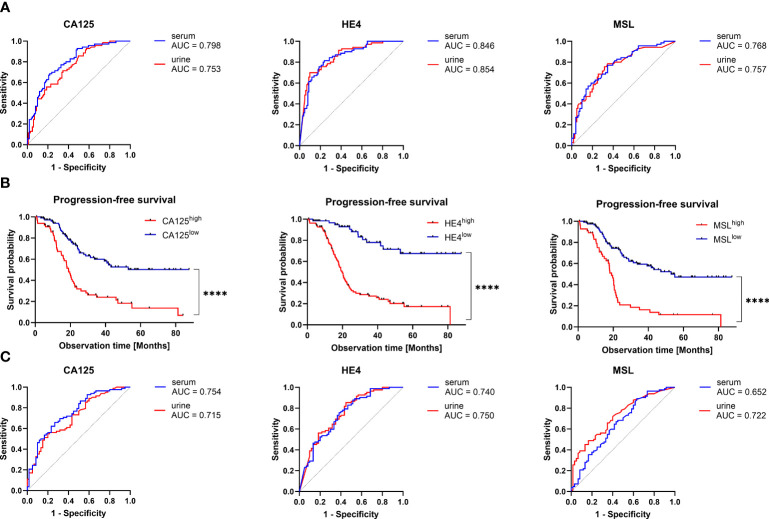
Predictive and prognostic value of CA125, HE4 and MSL in urine *vs.* serum. **(A)** Receiver operating characteristic (ROC)-curve analysis comparing the capacity of the indicated biomarkers to predict surgical outcome in matched urine *vs.* serum samples. The respective areas under the curve (AUC) are indicated. There were no statistically significant differences between the ROC-curves of each biomarker. **(B)** Kaplan-Meier analysis comparing PFS (relapse or death) in patients with high *vs.* low level of the indicated biomarkers. Optimal cut-off values (CA125: 23 U/mL, HE4: 16000pmol/L, 0.76 nmol/L) were calculated by ROC-curve analysis. P-values according to the Log-Rank (Mantel-Cox) test are indicated. ****P<0.0001. C) ROC-curve analysis comparing the capacity of the indicated biomarkers to predict PFS (relapse or death) before 48 months in matched urine *vs.* serum samples. There were no statistically significant differences between the ROC-curves of each biomarker.

We have previously described that the combination of pre-operative serum HE4 and CA125 was the optimal marker combination for blood-based prediction of surgical outcome, whereas this effect was mostly driven by HE4 (9). We subsequently compared the predictive performance of MSL, HE4 and CA125 for urinary- *vs.* serum-based prediction of surgical outcome. Interestingly, predictive performance of all three biomarkers was strikingly similar between urine and serum, with HE4 being the most accurate marker to predict surgical outcome in both fluid types (urine: AUC=0.854, 95%CI=0.797-0.911 *vs.* serum: AUC=0.846, 95%CI=0.788-0.905, respectively; **Figure 4A**; **Supplementary Table 1**). According to multivariate logistic regression analysis including all three biomarkers, we observed that HE4, but not MSL or CA125, was an independent predictor for surgical outcome, either in serum (β=0.702, 95%CI=0.432-0.972; p<0.0001) or in urine (β=0.924, 95%CI=0.622-1.226; p<0.0001; **Supplementary Table 2**).

In patients with early stage ovarian cancer (FIGO I-II; contributing to 20% in our present cohort), macroscopically complete tumor resection was typically achieved by surgical treatment. Optimal surgical outcome prediction by urinary HE4 was maintained, when considering exclusively advanced ovarian cancer patients with FIGO III-IV (AUC=0.836, 95%CI=0.769-0.904; **Supplementary Figures 1A–C**), suggesting that the inclusion of patients with early stage ovarian cancer did not confound our analysis.

Taken together, we report that urine-based prediction of surgical outcome is similarly efficient by urinary- *vs.* serum-based detection with HE4 providing the highest predictive capacity.

### 3.4 Urinary *vs*. serum-based prognostic modelling

We further analyzed the performance of urinary MSL, HE4 or CA125 levels to predict relapse or death before 48 months after primary diagnosis. Using optimal cut-offs, we performed Kaplan-Meier analyses, revealing that higher levels of urinary MSL (>0.76 nmol/L), HE4 (>16000 pmol/L) or CA125 (>23 U/mL), respectively, indicated poor prognosis (**Figure 4B**).

Relapse or death was predicted with AUC=0.722 (95%CI=0.638-0.805) for MSL, AUC=0.750 (95%CI=0.667-0.832) for HE4 and AUC=0.715 (95%CI=0.631-0.799) for CA125. Interestingly, performance to predict relapse or death before 48 months was virtually the same in matched serum samples (MSL: AUC=0.652 (95%CI=0.560-0.745); HE4: AUC=0.740 (95%CI=0.657-0.823); CA125: AUC=0.754 (95%CI=0.673-0.834); **Figure 4C**; **Supplementary Table 3**).

We performed univariate regression analysis, including pre-operative values of urinary MSL, HE4, CA125 and surgical outcome as the most established risk factors (6). In this setting, none of the three biomarkers, neither in serum nor urine, were independent prognostic factors (**Supplementary Table 4**). Nonetheless, by applying a more limited multivariate model (including MSL, HE4, CA125, age and BMI), we observed that urinary HE4 was an independent prognostic factor (p=0.0004; **Supplementary Table 5**). Using Akaike’s information criterion (AIC) estimation, we eventually determined the optimal setting for urinary prediction of relapse or death. The detection of HE4 alone was superior to all other biomarker combinations, indicated by the lowest AIC value (**Supplementary Table 6**).

In conclusion, we showed that the prognostic performance of pre-surgical MSL, HE4 or CA125 was similarly efficient in urinary- *vs.* serum-based detection, with urinary HE4 providing the strongest prognostic value for surgical outcome and relapse/death with an accuracy of 0.819 and 0.725 respectively (**Supplementary Tables 1**, **3**).

### 3.5 Effects of creatinine-based normalization of urinary biomarkers

**Figure 5:**
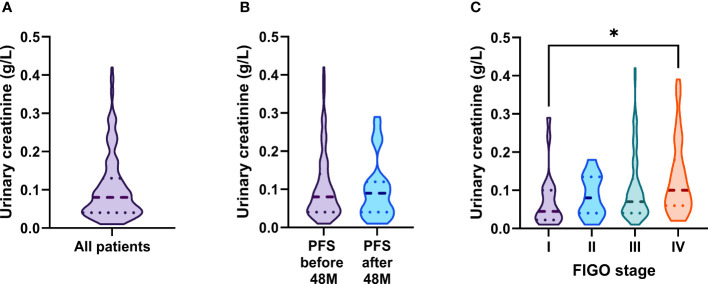
Distribution of urinary creatinine levels and its association with clinical parameters. **(A)** Distribution of urinary creatinine levels in ovarian cancer patients. Comparison of urinary creatinine levels across **(B)** patients with PFS (relapse or death) >/< 48 months and **(C)** the different FIGO-stages. The dashed line depicts the median and the dotted lines the first and third quartiles. Adjusted P-values according to the Kruskal-Wallis test with Dunn’s correction are indicated. *P<0.05.

As urinary creatinine (uCREA) excretion and turn-over rate is supposed to be constant over time, normalization of urinary biomarkers by uCREA may allow to compensate variations in urine flow rate across individuals (16). We were interested in the effect of uCREA-based normalization on the predictive/prognostic performance of urinary MSL, HE4 and CA125. Most of our patients (146/172, 84.9%) had uCREA levels in a narrow range between 0.01 and 0.19 g/L (**Figure 5A**) and there was no correlation between uCREA and serum creatinine (sCREA; r=-0.00679, R²=0.0046, p=0.3861), a marker of kidney function. While there was no significant difference in uCREA levels between patients with relapse/death before *vs.* after 48 months (**Figure 5B**), a slight difference in uCREA was observed between FIGO I *vs.* FIGO IV patients (**Figure 5C**). We subsequently re-analyzed our data, this time using uCREA-normalized values for MSL, HE4 and CA125. Normalization did not change any of the previously described key findings, on the predictive and prognostic capacity of the three urinary biomarkers (**Supplementary Figures 2**–**4**).

Taken together, we demonstrate that clinically relevant findings regarding urinary MSL, HE4 and CA125 were equally reproducible among raw *vs*. creatinine-normalized datasets.

## 4 Discussion

We have recently described a predictive/prognostic model for ovarian cancer, exploiting commonly available clinico-pathological factors and the three ovarian cancer serum biomarkers MSL, HE4 and CA125 (9). In the present study, we transferred this approach to a urine-based biomarker detection, demonstrating that serum levels of MSL, HE4 and CA125 correlated with matched urine samples and that prediction of surgical outcome or relapse/death by MSL, HE4 or CA125 was similarly efficient among urinary- *vs.* serum-based detection.

Based on their molecular weight, the investigated biomarkers showed crucial differences in their absolute urinary *vs.* serum concentrations, indicating different glomerular filtration rates. This supports the fact that the urinary level of a given biomarker is a function of its particular glomerular filtration rate and should carefully be considered for each urinary biomarker. We suppose that HE4, the smallest of the investigated proteins (40 kDa), most efficiently passes the glomerular filter. This could explain the high absolute HE4 concentrations in the urine and the observed strong correlation between i) serum *vs.* urinary HE4 and ii) serum HE4 *vs.* sCREA, an established marker of kidney function. Our findings are supported by previous studies, showing that serum HE4 but not CA125 levels are critically dependent on the glomerular filtration rate (17, 18).

We have previously reported that prognostic modelling using serum MSL, HE4 and CA125 is independent on *BRCA1/2* mutational status. Consistently, also urinary levels of these three biomarkers were unrelated to *BRCA1/2* status, suggesting that HRD-status will likely not confound the predictive/prognostic impact of these biomarkers. As a limitation of our study, we have included patients with primary diagnoses from 2013 to 2020, when HRD-testing was not yet implemented into routine clinical practice. HRD-testing has previously been implemented in clinical trials, such as PAOLA1 or PRIMA (2, 4), and has only recently (at the end of 2020) become available to patients outside of clinical trials. HRD, which is not limited to *BRCA1/2* mutations, is present in approximately 50% of high-grade serous ovarian tumors (19). Thus, *BRCA1/2* mutations, which are generally observed in only 22% of ovarian cancer patients (19) do not unmask all HRD cases.

As we previously reported that serum HE4 is the optimal biomarker for blood-based prediction of surgical outcome (9), we can conclude from our present study that urinary HE4 is fully equivalent for this purpose, allowing urinary analysis to identify ovarian cancer patients, in whom a macroscopically complete resection will not be possible. Those patients could be amenable for either primary chemotherapy without debulking surgery, which may reduce morbidity and mortality, or for neoadjuvant treatment. Whether urinary HE4 may also predict surgical outcome of interval debulking surgery, cannot be clarified in the present cohort and remains to be addressed by future investigation.

In our previous prognostic model, we showed that combined analysis of pre-operative serum CA125, post-operative serum MSL, post-operative serum HE4 and surgical outcome resulted in an optimal outcome prediction, which was superior to that of surgical outcome status, alone (9). However, pre-operative MSL, HE4 and CA125 levels in the present study, neither in serum nor in urine, were independent predictors of relapse/death when surgical outcome status was included into the multivariate regression model. This can be explained by the fact that our previous approach also enclosed post-operative serum samples, which further advanced the prognostic impact of this model. As a limitation of our study, only pre-operative urine samples were available, so the present study was restricted to only the pre-operative timepoint.

As a strength of our study, we clearly show that the individual prognostic relevance of MSL, HE4 and CA125 is virtually equivalent in serum vs. urine. This finding was particularly remarkable in case of CA125. CA125 values were considerably lower in urine than in serum (about 1000-fold). This is likely due to the size of the CA125 molecule (110kDa), resulting partial retention of CA125 by the glomerular filter in the kidney. It was all the more interesting that urinary CA125, besides its low absolute concentrations, showed similar prognostic capacity compared to serum CA125. Our work pioneers the concept that urinary detection of the indicated biomarkers provides a further dimension to be considered for prognostic modelling in ovarian cancer. Urinary biomarkers have several clinical advantages over serum biomarkers. Urine as the prototype non-invasive sample is a highly attractive fluid for biomarker discovery, since it is easily accessible without any trauma or pain on the patients and can be collected longitudinally and in a quantitative manner. Compared with serum or plasma, urinary peptides have several advantages for biomarker discovery, such as a high content of small and stable molecules. Furthermore, the urinary proteome is supposed reflect an informative “end product” of the abnormal protein metabolism in cancer (10).

The daily creatine turnover is around 2% of the total body creatine pool of which 98% is stored in the muscles (20, 21). This creatine turnover rate is known to be relatively constant in normal individuals (22) and urinary biomarker excretion should have a linear relationship with uCREA across individuals (16) allowing uCREA normalization to compensate variations in urine flow rate across individuals. Therefore, several previous studies on urinary biomarkers in ovarian cancer patients use uCREA normalization (23, 24). However, uCREA levels can also be confounded by several factors, such as muscle mass, physical activity, age or chronic kidney disease (25, 26) and there are also studies interpreting urinary biomarkers by raw datasets (27). We reported that clinically relevant findings of the urinary biomarkers MSL, HE4 and CA125 were stably reproducible among either raw or uCREA-normalized datasets. This indicates that uCREA normalization, at least for the proposed urinary biomarkers MSL, HE4 and CA125, is not an absolutely essential procedure, which may further simplify their potential use among routine laboratory diagnostics.

## 5 Conclusion

We report that the capacity of MSL, HE4 and CA125 to predict surgical outcome and relapse/death is equivalent between serum *vs.* urine-based detection. Urinary biomarkers, in particular HE4, may advance the spectrum of non-invasive biomarkers and could provide an additional dimension for prognostic modelling in ovarian cancer.

## Data availability statement

The raw data supporting the conclusions of this article will be made available by the authors, without undue reservation.

## Ethics statement

The studies involving humans were approved by Ethikkommission an der Technischen Universität Dresden, Technische Universität Dresden. The studies were conducted in accordance with the local legislation and institutional requirements. The participants provided their written informed consent to participate in this study.

## Author contributions

FS: Data curation, Formal analysis, Methodology, Software, Visualization, Writing – original draft, Writing – review & editing. DK: Data curation, Formal analysis, Methodology, Writing – review & editing. PW: Conceptualization, Supervision, Writing – review & editing. JK: Conceptualization, Data curation, Formal analysis, Project administration, Resources, Supervision, Writing – original draft, Writing – review & editing.
