# Supplementary material for: Urinary-based detection of MSL, HE4 and CA125 as an additional dimension for predictive and prognostic modelling in ovarian cancer
Source: Front Oncol. 2024 Jul 15;14:1392545. doi: 10.3389/fonc.2024.1392545 (PMC11284093; doi:10.3389/fonc.2024.1392545)
Supplement: Supplementary file 1 [file Image_1.pdf]

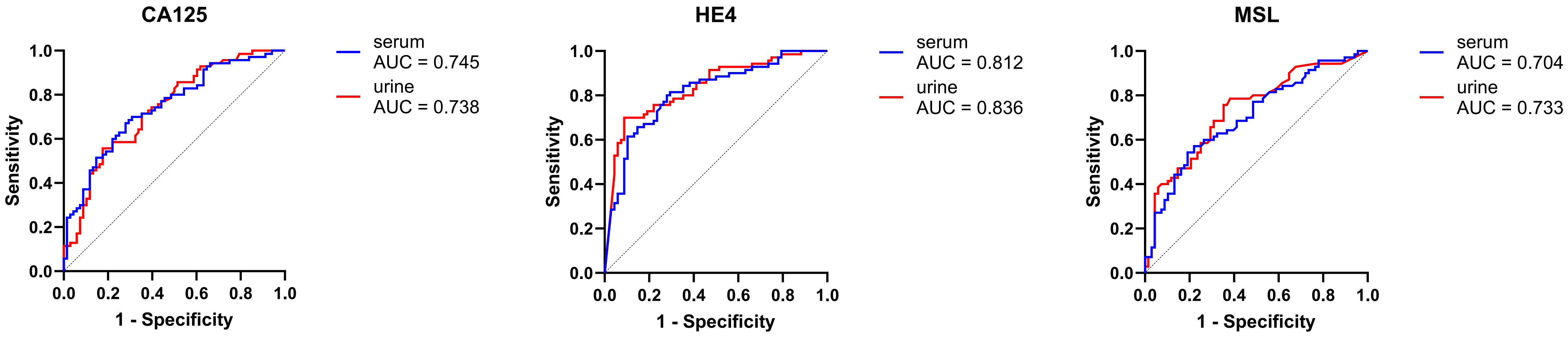

Supplementary Figure 1: Prediction of surgical outcome in advanced ovarian cancer patients (FIGO III-IV) by MSL, HE4 and CA125 in serum *vs.* urine. A) Receiver operating characteristic (ROC)-curve analysis comparing the capacity of the indicated biomarkers to predict surgical outcome in matched urine *vs*. serum samples. Only patients with advanced ovarian cancer (FIGO III-IV) were included into this analysis. The respective areas under the curve (AUC) are indicated.

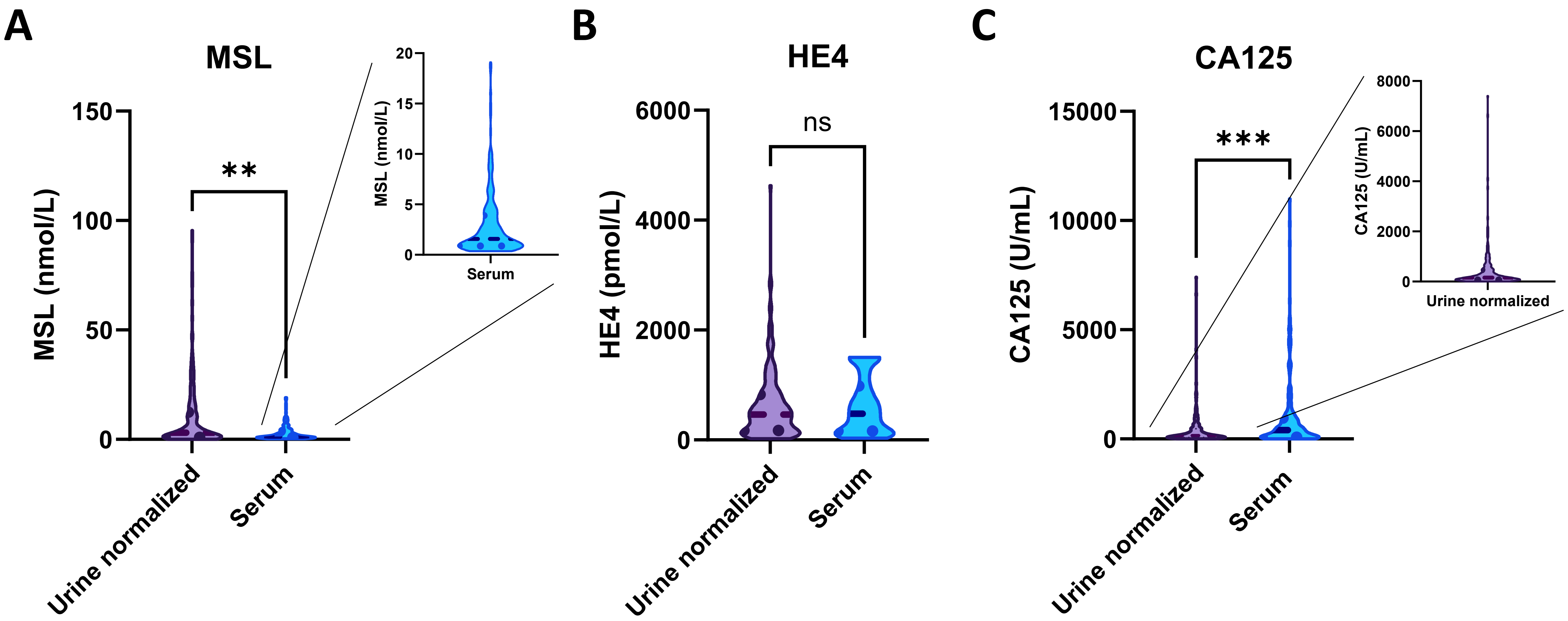

Supplementary Figure 2: Absolute concentrations of the designated biomarkers in urine *vs.* serum (re-analysis after normalization of urinary biomarkers). Violin plots depicting absolute concentrations of (**A**) HE4, (**B**) MSL and (**C**) CA125 in matched urine *vs.* serum samples. Urinary biomarkers have been normalized by urinary creatinine (uCREA). The dashed line depicts the median and the dotted lines the first and third quartiles. P-values according to the Mann-Whitney test are indicated. ** P <0.01, *** P <0.001.

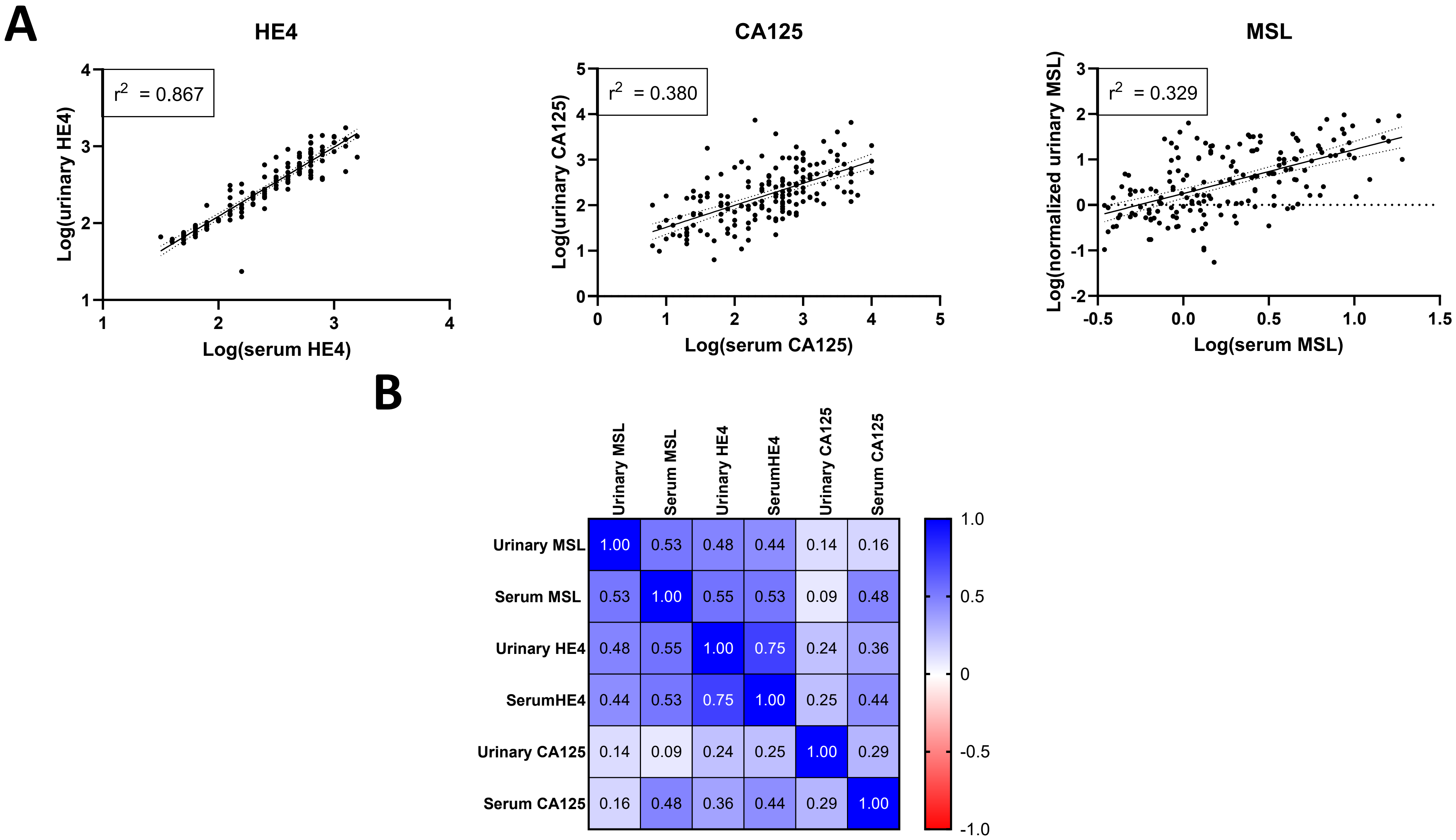

Supplementary Figure 3: Correlation of HE4, CA125 and MSL in urine *vs.* serum (re-analysis after normalization of urinary biomarkers). Correlation metrics of the three biomarkers are reported according to (**A**) linear regression analysis and (**B**) Pearson correlation. Urinary biomarkers have been normalized by urinary creatinine (uCREA).

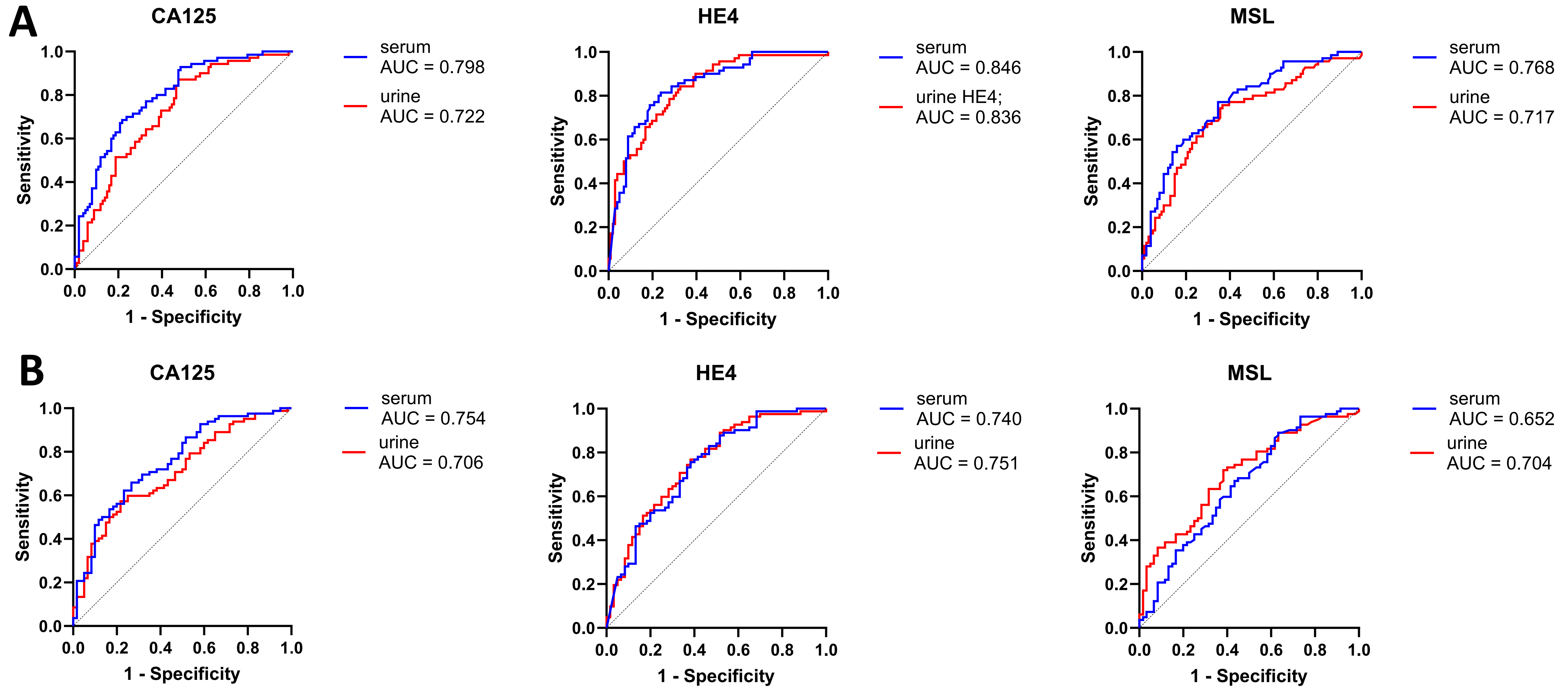

Supplementary Figure 4: Predictive value of CA125, HE4 and MSL in urine *vs.* serum (re-analysis after normalization of urinary biomarkers). A) Receiver operating characteristic (ROC)-curve analysis comparing the capacity of the indicated biomarkers to predict surgical outcome in matched urine *vs.* serum samples. **B**) ROC-analysis comparing the capacity of the indicated biomarkers to predict PFS (relapse or death) before 48 months in matched urine *vs*. serum samples. The respective areas under the curve (AUC) are indicated. Urinary biomarkers have been normalized by urinary creatinine (uCREA).
