# Supplementary material for: Urinary-based detection of MSL, HE4 and CA125 as an additional dimension for predictive and prognostic modelling in ovarian cancer
Source: Front Oncol. 2024 Jul 15;14:1392545. doi: 10.3389/fonc.2024.1392545 (PMC11284093; doi:10.3389/fonc.2024.1392545)
Supplement: Supplementary file 2 [file Table_1.docx]

Supplementary Tables

**Supplementary Table 1: Capacity of MSL, HE4 and CA125 to predict surgical outcome in serum *vs.* urine (ROC-curve analysis)**

| **Marker** | **AUC** | **Standard error** | **Lower bound (95%)** | **Upper bound (95%)** | **Optimal Threshold** | **Sensitivity** | **Lower bound (95%)** | **Upper bound (95%)** | **Specificity** | **Lower bound (95%)** | **Upper bound (95%)** | **Sensitivity+ Specificity** | **Accuracy** |  |
| --- | --- | --- | --- | --- | --- | --- | --- | --- | --- | --- | --- | --- | --- | --- |
| **Serum CA125** | 0.798 | 0.034 | 0.732 | 0.863 | 515.9 | 0.686 | 0.569 | 0.782 | 0.782 | 0.691 | 0.852 | 1.468 | 0.743 |  |
| **Urinary CA125** | 0.753 | 0.037 | 0.681 | 0.825 | 26.6 | 0.557 | 0.441 | 0.667 | 0.812 | 0.723 | 0.877 | 1.369 | 0.708 |  |
| **Serum HE4** | 0.846 | 0.030 | 0.788 | 0.904 | 497.7 | 0.814 | 0.706 | 0.889 | 0.762 | 0.670 | 0.835 | 1.577 | 0.784 |  |
| **Urinary HE4** | 0.854 | 0.029 | 0.797 | 0.911 | 68603 | 0.700 | 0.584 | 0.795 | 0.901 | 0.825 | 0.947 | 1.601 | 0.819 |  |
| **Serum MSL** | 0.768 | 0.036 | 0.697 | 0.839 | 1.43 | 0.771 | 0.659 | 0.855 | 0.653 | 0.556 | 0.739 | 1.425 | 0.702 |  |
| **Urinary MSL** | 0.757 | 0.038 | 0.682 | 0.831 | 0.23 | 0.757 | 0.644 | 0.843 | 0.683 | 0.587 | 0.766 | 1.440 | 0.713 |  |
| *AUC = area under the curve; All AUCs showed a P-value <0.0001 vs. diagonal line (random classifier)* | | | | | | | | | | | | | | |

**Supplementary Table 2: Capacity of MSL, HE4 and CA125 to predict surgical outcome in serum *vs.* urine (multivariate logistic regression analysis)**

|  | β coefficient | Standard error | Wald Chi-Square | Pr > Chi² | Wald Lower bound (95%) | Wald Upper bound (95%) | Odds ratio (exp β) |
| --- | --- | --- | --- | --- | --- | --- | --- |
| Serum MSL | 0.163 | 0.251 | 0.420 | 0.517 | -0.329 | 0.654 | 1.454 |
| Serum HE4 | 0.702 | 0.138 | 25.946 | **< 0.0001** | 0.432 | 0.972 | 5.031 |
| Serum CA125 | 0.359 | 0.184 | 3.823 | 0.051 | -0.001 | 0.719 | 2.287 |
| Urinary MSL | -0.080 | 0.121 | 0.440 | 0.507 | -0.318 | 0.157 | 0.831 |
| Urinary HE4 | 0.924 | 0.154 | 35.963 | **< 0.0001** | 0.622 | 1.226 | 8.395 |
| Urinary CA125 | -0.039 | 0.116 | 0.113 | 0.737 | -0.267 | 0.189 | 0.914 |

*β coefficient = standardized coefficient value of logistic regression*

**Supplementary Table 3: Capacity of MSL, HE4 and CA125 (serum *vs.* urine) to predict relapse or death before 48 months (ROC-curve analysis)**

| **Marker  (for PFS <48M)** | **AUC** | **Standard error** | **Lower bound (95%)** | **Upper bound (95%)** | **Optimal threshold** | **Sensitivity** | **Lower bound (95%)** | **Upper bound (95%)** | **Specificity** | **Lower bound (95%)** | **Upper bound (95%)** | **Sensitivity + Specificity** | **Accuracy** |  |
| --- | --- | --- | --- | --- | --- | --- | --- | --- | --- | --- | --- | --- | --- | --- |
| **Serum CA125** | **0.753** | 0.041 | 0.673 | 0.834 | **493.6** | **0.659** | 0.550 | 0.752 | **0.733** | 0.609 | 0.829 | **1.392** | **0.690** |  |
| **Urinary CA125** | **0.715** | 0.043 | 0.631 | 0.799 | **23.1** | **0.549** | 0.441 | 0.652 | **0.800** | 0.680 | 0.883 | **1.349** | **0.655** |  |
| **Serum HE4** | **0.740** | 0.042 | 0.657 | 0.822 | **413.4** | **0.756** | 0.652 | 0.836 | **0.617** | 0.490 | 0.729 | **1.373** | **0.697** |  |
| **Urinary HE4** | **0.750** | 0.042 | 0.668 | 0.831 | **16109** | **0.854** | 0.759 | 0.915 | **0.550** | 0.425 | 0.669 | **1.404** | **0.725** |  |
| **Serum MSL** | **0.652** | 0.047 | 0.560 | 0.745 | **0.92** | **0.890** | 0.802 | 0.943 | **0.367** | 0.256 | 0.494 | **1.257** | **0.669** |  |
| **Urinary MSL** | **0.722** | 0.042 | 0.639 | 0.805 | **0.76** | **0.488** | 0.383 | 0.594 | **0.833** | 0.717 | 0.908 | **1.321** | **0.634** |  |
| *AUC = area under the curve, FIGO = Fédération Internationale de Gynécologie et d'Obstétrique;* *All AUCs showed a P-value <0.01 vs. diagonal line (random classifier)* | | | | | | | | | | | | | | |

**Supplementary Table 4: Capacity of MSL, HE4 and CA125 (serum *vs.* urine) to predict relapse or death before 48 months (multivariate logistic regression analysis)**

|  | β coefficient | Standard error | Wald Chi-Square | Pr > Chi² | Wald Lower bound (95%) | Wald Upper bound (95%) | odds ratio (expβ) |
| --- | --- | --- | --- | --- | --- | --- | --- |
| Serum MSL | -0.225 | 0.189 | 1.419 | 0.234 | -0.596 | 0.145 | 0.595 |
| Serum HE4 | 0.170 | 0.150 | 1.289 | 0.256 | -0.124 | 0.464 | 1.480 |
| Serum CA125 | 0.471 | 0.318 | 2.197 | 0.138 | -0.152 | 1.094 | 2.958 |
| Macroscopic complete resection | 0.000 | 0.000 |  |  |  |  | 1.000 |
| Any residual tumor | 0.603 | 0.131 | 21.049 | < 0.0001 | 0.345 | 0.861 | 4.010 |
| Urinary MSL | 0.195 | 0.210 | 0.862 | 0.353 | -0.217 | 0.608 | 1.568 |
| Urinary HE4 | -0.014 | 0.177 | 0.006 | 0.937 | -0.362 | 0.334 | 0.968 |
| Urinary CA125 | 0.407 | 0.293 | 1.925 | 0.165 | -0.168 | 0.982 | 2.553 |
| Macroscopic complete resection | 0.000 | 0.000 |  |  |  |  | 1.000 |
| Any residual tumor | 0.585 | 0.137 | 18.149 | < 0.0001 | 0.316 | 0.854 | 3.845 |

*β coefficient = standardized coefficient value of logistic regression*

**Supplementary Table 5: Capacity of urinary MSL, HE4 and CA125 to predict relapse or death before 48 months (multivariate logistic regression analysis)**

| **Source** | **DF** | **Chi-square (Wald)** | **Pr > Wald** | **Chi-square (LR)** | **Pr > LR** |
| --- | --- | --- | --- | --- | --- |
| **Urinary MSL** | 1 | 1.089 | 0.297 | 1.474 | 0.225 |
| **Urinary HE4** | 1 | 11.827 | 0.001 | 12.964 | **0.0004** |
| **Urinary CA125** | 1 | 0.144 | 0.705 | 0.150 | 0.699 |
| **Age ≥60;<60** | 1 | 0.131 | 0.718 | 0.131 | 0.718 |
| **BMI ≥30;<30** | 1 | 0.158 | 0.691 | 0.158 | 0.691 |
| *BMI = Body mass index, DF = degrees of freedom, Pr = probability distribution associated to the binary distribution (event 1/0), LR = Likelihood ratio* | | | | | |

**Supplementary Table 6: Selection of the optimal biomarker combination for surgical outcome prediction using Akaike’s information criterion (AIC) estimation**

| **Nr. of variables** | **Variables** | **-2 Log(Likelihood)** | **Pr > LR** | **Pr > Score** | **Pr > Wald** | **Akaike's AIC** |
| --- | --- | --- | --- | --- | --- | --- |
| **1** | **Urinary HE4** | 199.192 | 0.000 | 0.000 | 0.000 | **205.192** |
| **2** | **Urinary MSL/ Urinary HE4** | 197.596 | 0.000 | 0.000 | 0.000 | **205.596** |
| **3** | Urinary MSL / Urinary HE4 / Urinary CA125 | 197.354 | 0.000 | 0.000 | 0.000 | 207.354 |
| **4** | Urinary MSL / Urinary HE4 / Urinary CA125 / BMI ≥30;<30 | 197.231 | 0.000 | 0.000 | 0.000 | 209.231 |
| **5** | Urinary MSL / Urinary HE4 / Urinary CA125 / Age ≥60;<60 / BMI ≥30;<30 | **197.100** | 0.000 | 0.000 | 0.000 | 211.100 |
| *The lowest Akaike information criterion (AIC) scores indicate the best fitting model using fewer independent variables. BMI = body mass index, Pr = probability distribution associated to the binary distribution (event 1/0).* | | | | | | |
|  | | | | | | |
